# Torilin Inhibits Inflammation by Limiting TAK1-Mediated MAP Kinase and NF-*κ*B Activation

**DOI:** 10.1155/2017/7250968

**Published:** 2017-02-20

**Authors:** Mehari Endale, Tae-Hwan Kim, Yi-Seong Kwak, Na-Mi Kim, Seung-Hyung Kim, Jae Youl Cho, Bong-Sik Yun, Man-Hee Rhee

**Affiliations:** ^1^Division of Neonatology and Pulmonary Biology, Cincinnati Children's Hospital Research Foundation, Cincinnati, OH, USA; ^2^College of Veterinary Medicine, Kyungpook National University, Daegu 702-701, Republic of Korea; ^3^Research and Development Headquarters, Korea Ginseng Corporation, Daejon 305-805, Republic of Korea; ^4^Institute of Traditional Medicine & Bioscience, Daejeon University, Daejeon 300-716, Republic of Korea; ^5^Department of Genetic Engineering, Sungkyunkwan University, Suwon 440-746, Republic of Korea; ^6^College of Environmental & Bioresource Sciences, Chonbuk National University, Iksan 570-752, Republic of Korea

## Abstract

Torilin, a sesquiterpene isolated from the fruits of* Torilis japonica,* has shown antimicrobial, anticancer, and anti-inflammatory properties. However, data on the mechanism of torilin action against inflammation is limited. This study aimed at determining the anti-inflammatory property of torilin in LPS-induced inflammation using in vitro model of inflammation. We examined torilin's effect on expression levels of inflammatory mediators and cytokines in LPS-stimulated RAW 264.7 macrophages. The involvement of NF-kB and AP-1, MAP kinases, and adaptor proteins were assessed. Torilin strongly inhibited LPS-induced NO release, iNOS, PGE_2_, COX-2, NF-*α*, IL-1*β*, IL-6, and GM-CSF gene and protein expressions. In addition, MAPKs were also suppressed by torilin pretreatment. Involvement of ERK1/2, P38^MAPK^, and JNK1/2 was further confirmed by PD98059, SB203580, and SP600125 mediated suppression of iNOS and COX-2 proteins. Furthermore, torilin attenuated NF-kB and AP-1 translocation, DNA binding, and reporter gene transcription. Interestingly, torilin inhibited TAK1 kinase activation with the subsequent suppression of MAPK-mediated JNK, p38, ERK1/2, and AP-1 (ATF-2 and c-jun) activation and IKK-mediated I-*κ*B*α* degradation, p65/p50 activation, and translocation. Together, the results revealed the suppression of NF-*κ*B and AP-1 regulated inflammatory mediator and cytokine expressions, suggesting the test compound's potential as a candidate anti-inflammatory agent.

## 1. Introduction

The study of such inflammatory responses including inflammatory mediators and cytokines activation is best characterized using suitable macrophage cell-lines including RAW 264.7 cells as models. In macrophages, LPS activates Toll-like receptor-4 (TLR4) and the activated receptor recruits the adaptor proteins (e.g., MyD88 or TRAM) and initiates MyD88 dependent or independent signaling pathways. MyD88 recruits and associates with two IL-1R-associated kinases (IRAK4 and IRAK1) leading to TNF receptor associated factor-6 (TRAF6) activation [1]. The activated TRAF6 is then ubiquitinated and recruited to the TGF*β*-activated kinase 1 (TAK1)-TGF*β*-activated kinase-1 binding protein 1 and 2 (TAB1/2) complex via binding to TAB2 [2]. This promotes the activation of TAK1, which in turn activates the canonical NF-*κ*B [2] and the MAP kinases signaling pathways [3]. TAK1-TAB1/2 complex is thus a common route for the I*κ*B kinase (IKK) and for MAPKs pathways (ERK1/2, JNK, and p38) [4]. These signaling pathways in turn activate a variety of transcription factors including NF-kB (p50/p65) and AP-1 (c-Fos/c-Jun) that coordinate the induction of many genes encoding inflammatory mediators [5]. These mediators such as iNOS, COX-2, and cytokines (TNF-*α*, IL-1*β*, IL-6, and GM-CSF) are the common inflammatory mediators transcriptionally regulated by the above indicated transcription factors [6]. Therefore, targeting these transcription factors, adaptor proteins, protein kinases, inflammation mediators, or cytokines themselves by natural products or their derivatives can be an effective strategy for immunosuppression [7].

Natural products are reported to be better candidates of therapeutics due to their chemical diversity, structural complexity, affordability, lack of substantial toxicity, and inherent biologic activities [8]. Salminen et al. further indicated that a number of natural products have demonstrated NF-*κ*B-inhibitory activities such as attenuation of IKK activation, I*κ*B degradation, NF-*κ*B nuclear translocation, and DNA binding [9]. A large body of evidences also indicated anti-inflammatory effects of natural products against MAP kinases. Modulation of NF-*κ*B and MAP kinases signaling pathways is therefore a principal target to alleviate inflammatory diseases including arthritis [10]. Recently, however, the demand for anti-inflammatory agents that can treat inflammation and arthritis but remain relatively free of side effects in long term treatment increases.

A large number of plant-derived compounds including natural terpenoids as powerful inhibitors of NF-kB and MAP kinases are reported to be suppressive agents of inflammation and cancer [9, 11]. Evidently, a number of terpenoids are reported to affect some upstream targets as they inhibit the NF-kB and MAPK pathways simultaneously and further potentiate the suppression of inflammatory responses [9, 12]. Sesquiterpenes are most widely published class of natural products cited as inhibitors of NF-*κ*B. For instance, parthenolide, a sesquiterpenes, is reported to inhibit NF-*κ*B via inhibition of I*κ*B*α*/I*κ*B*β* degradation [13] and IKK*β* activation [14] and modification of NF-*κ*B via alkylation [15–17]. However, information on the mechanism of anti-inflammatory property of torilin, another sesquiterpene, is limited.

Torilin (11-acetoxy-8-angeloyl-4-guaien-3-one), a guaiane-type sesquiterpene angelate, is isolated from the fruits of the plant* Torilis japonica* [18]. Previous studies on torilin indicated that the compound exhibits antiprotozoal [19] and antimicrobial [20] effects. Besides, it is reported to reverse multidrug-resistance [21] and show antiangiogenic as well as anti-invasive properties [22, 23].

Although few reports have been documented on the anti-inflammatory potential of torilin [24, 25], studies detailing the mechanism of action behind its anti-inflammatory effect using macrophage cells line (suitable models for studying inflammatory responses) are limited. Since torilin has been shown to possess anti-inflammatory activities in vitro and in vivo [24, 25], we previously have reported that torilin modifies inflammatory cell and cytokine imbalances with the attenuation of the severity of arthritis in mouse model of rheumatoid arthritis [26]. However, its molecular mechanism of action against inflammatory responses has not been reported yet. The aim of this study was therefore to examine the upstream events in the anti-inflammatory property of torilin and to elucidate its underlying mechanisms of action. Here, we report that torilin markedly inhibited inflammatory mediators and cytokines via inhibition of TAK1-mediated MAPK, AP-1, and NF-kB activation.

## 2. Materials and Methods

Primary antibodies for iNOS, COX-2, *β*-actin, PARP, phospho-PI3K p85, PI3K, Akt, phospho-Akt, NF-*κ*B, phospho-NF-*κ*B, I*κ*B-*α*, phospho-I*κ*B-*α*, IKK*β*, phospho-IKK*α*/*β*, phospho-p38, p38^MAPK^, phospho-JNK, JNK, phospho-ERK1/2, ERK1/2, MKK4, MKK6, MyD88, IRAK1, TRAF6, phosphor-TAK1, TAK1, phospho-c-fos, phosphor-c-jun, phosphor-ATF2, IL-1*β*, TNF*α*, and horseradish peroxidase-conjugated secondary antibody were from Cell Signaling Technology (Danvers, MA, USA). SB203580, SP600125, and PD98059 were from Sigma-Aldrich (St. Louis, MO). Consensus oligonucleotides for NF-*κ*B and AP-1 were obtained from Santa Cruz Biotechnology (Santa Cruz, CA). *γ*^−32^ P-Labeled ATP was purchased from ICN (Costa Mesa, CA). A prostaglandin E_2_ EIA kit was from Enzo life Sciences (Ann Arbor, MI, USA). Easy Blue™ RNA extraction kit was from iNtRON, Korea. Millipore MILLIPLEX™ Mouse Cytokine/Chemokine enzyme-linked immunosorbent assay kit was from Millipore Corp. (St. Charles, MO). All materials, equipment, and biotinylated marker proteins for gel electrophoresis were from Bio-Rad. All other chemicals were purchased from Sigma-Aldrich (St. Louis, MO) unless otherwise stated. Torilin (98%) was prepared as previously indicated in [26] and dissolved in dimethyl sulfoxide and freshly diluted in culture media for all experiments.

### 2.1. Cell Culture

RAW 264.7 murine macrophages, obtained from the American cell collection (ATCC TIB71), were grown in Dulbecco's modified Eagle's medium (DMEM) (Invitrogen, Carlsbad, CA), supplemented with 10% fetal bovine serum, 2 mM L-glutamine, 100 UmL^−1^ penicillin, and 100 *µ*gmL^−1^ streptomycin. They were incubated under endotoxin-free conditions at 37°C in a 5% CO_2_ humidified air incubator.

### 2.2. Cell Viability

The RAW 264.7 cells were plated at a density of 5 × 10^4^ cells/well in a 96-well plate for 24 h. To determine any potential cytotoxic effect of the test compound, cells were treated with torilin or vehicle before incubation for 48 h. The 3-(4,5-dimethylthiazol-2-yl)-2,5-diphenyltetrazolium bromide (MTT, 0.5 mgmL^−1^) was added and cells were incubated for 4 h. The media were then removed, and produced formazan crystals in the wells were dissolved by addition of 200 mL dimethyl sulfoxide (DMSO). Absorbance was measured at 540 nm using Synergy HT Multi-Model Microplate Reader (BioTek Instrument, Winooski, USA). Cell viability (% control) was defined relative to untreated control cells.

### 2.3. Nitrite (NO) Production

RAW 264.7 macrophages were plated in 96-well plates (2 × 10^5^ cells/well) and incubated overnight. The cells were treated with torilin or vehicle 30 min before LPS stimulation for 24 h. NO production was determined via quantitation of nitrite levels in cell culture supernatants according to the Griess reaction with the absorbance measured at 540 nm.

### 2.4. Enzyme-Linked Immunosorbent Assay

RAW 264.7 cells were preincubated with torilin for 30 min before LPS stimulation for 24 h, and cytokine contents in the culture medium were measured by ELISA using anti-mouse TNF-*α*, IL-1*β*, IL-6, and GM-CSF antibodies and biotinylated secondary antibodies following the manufacturer's instruction (Millipore MILLIPLEX™ Mouse Cytokine/Chemokine kit (Millipore Corp., St. Charles, MO, USA)). In addition, PGE_2_ contents in the culture medium were measured using prostaglandin E_2 _kit according to the manufacturer's instruction (Enzo life Sciences, Ann Arbor, MI, USA).

### 2.5. RNA Isolation and Reverse Transcription PCR

Total cellular RNA from 3 × 10^6^ RAW 264.7 macrophages treated with torilin or vehicle was extracted as described previously [27] using Easy Blue kits (iNtRON Biotechnology, Korea) according to the manufacturer's instructions and stored at −70°C until use. Briefly, 1 *µ*g RNA was annealed with poly(dT)_18_ for 10 min at 70°C and cooled for 5 min on ice, reverse transcribed using reverse transcription (RT) premix (Bioneer) in 20 *µ*l of reaction mixture containing 5x buffer (10 mM dNTP, 0.1 mM dithiothreitol, and 2 U of murine leukemia virus reverse transcriptase), and run for 90 min at 42.5°C using a thermal cycler. The reactions were terminated at 95°C for 5 min to inactivate the reverse transcriptase. The reverse transcription polymerase chain reaction (RT-PCR) was performed using aliquots of cDNA obtained from RT reaction in a PCR premix (Bioneer) containing a 10x buffer [10 mM Tris·HCl (pH 8.3), 50 mM KCl, 0.1% Triton X-100, 0.25 mM dNTP, 25 mM MgCl_2_, and 1 U of Taq polymerase]. Amplification conditions were 5 min before denaturation at 95°C followed by 30–35 cycles consisting of denaturation at 95°C, annealing at 55–60°C, and elongation at 72°C for 45 second each with final extension for 10 min at 72°C. The PCR products were electrophoresed in 1.3% agarose gel stained with ethidium bromide and visualized using Eagle Eyes image analysis software (Stratagene, La Jolla, CA). The intensity of band densities for iNOS, COX-2, TNF-*α*, IL-1*β*, IL-6, and GM-CSF mRNA expression levels were normalized for the corresponding GAPDH and ratios were compared. The sequence of oligonucleotides used was as follows:* iNOS*: (forward-5′-GTG CTG CCT CTG GTC TTG CAA GC-3′, reverse-5′-AGG GGC AGG CTG GGA ATT CG-3′);* COX-2*: (forward-5′-TCT CAG CAC CCA CCC GCT CA-3′, reverse-5′-TCT CAG CAC CCA CCC GCT CA-3′);* IL-1β*: (forward-5′-TGC TTC CAA ACC TTT GAC CTG GGC-3′, reverse-5′-CAG GGT GGG TGT GCC GTC TTT C-3′);* TNF-α*: (forward-5′-CCT GTA GCC CAC GTC GTA GC-3′, reverse-5′-TTG ACC TCA GCG CTG AGT TG-3′);* IL-6*: (forward-5′-GCT GGA GTC ACA GAA GGA GTG GC-3′, reverse-5′-GGC ATA ACG CAC TAG GTT TGC CG-3′);* GM-CSF: *(forward-5′-ACT CTG CTC ACG AAG GAA CTC AGC-3′, reverse-5′-CAC AGC TCG GAA GAG CAT CGC A-3′);* GAPDH*: (forward-5′-CAC TCA CGG CAA ATT CAA CGG C-3′, reverse-5′-CCT TGG CAG CAC CAG TGG ATG CAG G-3′).

### 2.6. Immunoblotting

RAW 264.7 macrophages were washed with PBS and lysed in standard lysis buffer [20 mM Tris-HCl (pH 7.5), 1% Triton X-100, 137 mM NaCl, 10% glycerol, 2 mM EDTA, 1 mM sodium orthovanadate, 25 mM *β*-glycerophosphate, 2 mM sodium pyrophosphate, 1 mM phenylmethylsulfonyl fluoride and 1 mgmL^−1^ leupeptin, 2 mgmL^−1^ aprotinin, and 1 mgmL^−1^ pepstatin A]. Lysates were centrifuged at 10,000 ×g for 10 min and supernatant was stored at −70°C until use. Protein was then measured using Bicinchoninic Acid (BCA, Pierce, USA) protein assay kit with bovine serum albumin used as a standard. Equal amounts of protein (30–40 *µ*g/lane) were loaded with sample buffer (250 mM Tris-HCl, pH 6.8, 0.5 M DTT, 10% SDS, 50% glycerol, 5%  *β*-mercaptoethanol, and 0.5% bromophenol blue) and electrophoresed on 8–12% SDS-polyacrylamide gel under standard conditions and electroblotted to polyvinylidene difluoride membranes (PVDF) (Millipore Co., MA, USA) in 20% methanol transfer buffer. The membranes were washed three times with Tris-buffered saline containing 0.01% Tween-20 (TBS-T) and blocked with 5% nonfat milk, washed, and incubated with primary antibodies diluted (1 : 1000) overnight at 4°C. The membranes were then incubated with secondary antibodies conjugated with horseradish peroxidase for 1 h at room temperature. Scanning densitometry of the immunoblots was performed with an Image Scan and Analysis System (Alpha-Innotech, San Leandro, CA, USA). The area of each lane was integrated using the software Alpha Ease version 5.5 (Alpha-Innotech) followed by background subtraction.

### 2.7. Immunoprecipitation and In Vitro TAK1 Kinase Assays

Cell lysates containing equal amounts of protein (500 *μ*g) from RAW 264.7 (10^7^ cells/ml) treated with torilin or vehicle 30 min before LPS stimulation for 15 min were precleared with 10 *μ*L protein A-coupled Sepharose magnetic beads (10% v/v; ELPIS Biotech, Korea) for 1 h at 4°C. Precleared samples were incubated with 3 *μ*L anti-TAK1 antibody overnight at 4°C. Immune complexes were mixed with 10 *μ*L protein A-coupled Sepharose magnetic beads (10% v/v) and incubated for 4 h at 4°C. The immune-precipitates were then washed 5 times with immunoprecipitation (IP) buffer (20 mM Tris-HCl, pH 7.4; 2 mM EDTA, 2 mM EGTA, 50 mM *β*-glycero-phosphate, 5 mM NaF, 1 mM sodium orthovanadate, 1% Triton X-100, 10% glycerol, 10 *μ*g/ml aprotinin, 10 *μ*g/ml pepstatin, 1 mM PMSF, 1 mM benzimide, and 2 mM hydrogen peroxide). After washing, the beads were boiled in Laemmli sample buffer (2% sodium dodecyl sulfate [SDS], 1%  *β*-mercaptoethanol, 0.008% bromophenol blue, 80 mM Tris pH 6.8, and 1 mM EDTA) and the proteins were resolved by SDS-PAGE and transferred to PVDF membranes, blocked, and probed with anti-p-TAK1, TAK1, p-IKK*α*/*β*, IKK, p-MKK4, MKK4, anti-TLR4, and *β*-actin antibodies. Then, immunoblots were visualized as indicated above.

For evaluating TAK1 kinase activities following immunoprecipitation, the beads were washed in IP buffer and then in kinase reaction buffer [100 mM Tris-HCl, pH 7.2; 125 mM Mg (C_2_H_3_O_2_)_2_; 25 mM MnCl_2_; 2 mM EGTA; 0.25 mM sodium orthovanadate; and 2 mM DTT] and then resuspended in kinase buffer containing 1 *µ*m ATP and 12.5 *µ*Ci of *γ*^−32^. After 5 min of incubation at 30°C, the reaction was terminated by lysis buffer.

### 2.8. Preparation of Nuclear Extracts

Prior to nuclear extraction, cells (5 × 10^6^/well) were washed twice with ice-cold PBS centrifuged and the pellet was suspended by adding 400 *µ*l of buffer A [10 mM HEPES (pH 7.9), 10 mM KCl, 2 mM MgCl_2_, 0.1 mM EDTA, 1 mM dithiothreitol and 0.5 mM phenylmethylsulfonyl fluoride (PMSF), 2 *µ*gmL^−1^ of leupeptin, aprotinin, and pepstatin]. After centrifugation at 10,000 ×g for 5 min, pellet was resuspended with buffer A and 5% Nonidet-P40 and centrifuged at 13,000 ×g for 2 min and the supernatant was used as cytosolic extract. The pellets containing crude nuclei were resuspended in 400 *µ*l buffer A centrifuged at 10,000 ×g for 5 min twice. The pellets were again resuspended with buffer B containing 10 mM HEPES [(pH 7.9), 300 mM NaCl, 0.1 mM EDTA, 1 mM DTT and 1 mM PMSF, 5 *µ*gmL^−1^ leupeptin, aprotinin, and pepstatin each and 10% glycerol] and incubated with rocking for 20 min on ice. The samples were centrifuged at 15,800 ×g for 10 min to obtain the supernatant containing nuclear extracts. Protein concentration of the supernatant was determined.

### 2.9. Electrophoretic Mobility Shift Assay

RAW 264.7 macrophages (3 × 10^6^ cells) were treated with torilin or vehicle and stimulated with LPS for 45 min, washed, scraped into 0.5 ml cold PBS, and pelleted by centrifugation. Cytosolic and nuclear protein fractions were extracted using active motif nuclear extraction kit (Carlsbad, CA, USA). Binding reactions were performed at 37°C for 15 min in 20 *µ*l of reaction buffer containing [10 mM Tris-HCl, pH 7.5, 50 mM NaCl, 1 mM EDTA, 10% glycerol, 1 *µ*g poly(dI-dC), 1 mM dithiothreitol, and 30,000 cpm ^32^P-labeled oligonucleotide probes] for NF-*κ*B and AP-1. DNA-protein complexes were separated from unbound DNA probe on native 6% polyacrylamide gels at 75V in 0.5x TBE buffer and then transferred to nylon membrane.

### 2.10. Transient Transfection Luciferase Assay

Transient transfections of RAW 264.7 cells with MyD88, NF-*κ*B, and AP-1 promoter firefly luciferase construct were performed in triplicate in 24-well plates using Lipofectamine™ 2000 (Invitrogen, CA, USA). The reporter activity was measured using a luciferase assay system according to the manufacturer's instructions. Briefly, 6 hr after treatment, cells were washed twice with ice-cold PBS (pH 7.4) and lysed by adding 200 *μ*l of 1x reporter lysis buffer (Promega). After centrifugation at 12,000 ×g for 10 min at 4°C, the supernatant was analyzed for luciferase activity and normalized to *β*-galactosidase activity.

### 2.11. Statistical Analysis

Data were analyzed by one-way analysis of variance with Dunnett's post hoc test. Experiments represent at least 4 independent replications in triplicate. Values are means ± SE and treatment groups were compared by* t*-test. *P* < 0.05 was considered statistically significant.

## 3. Result

### 3.1. Torilin Inhibits LPS-Induced Inflammatory Mediator and Cytokine Expressions

**Figure 1:**
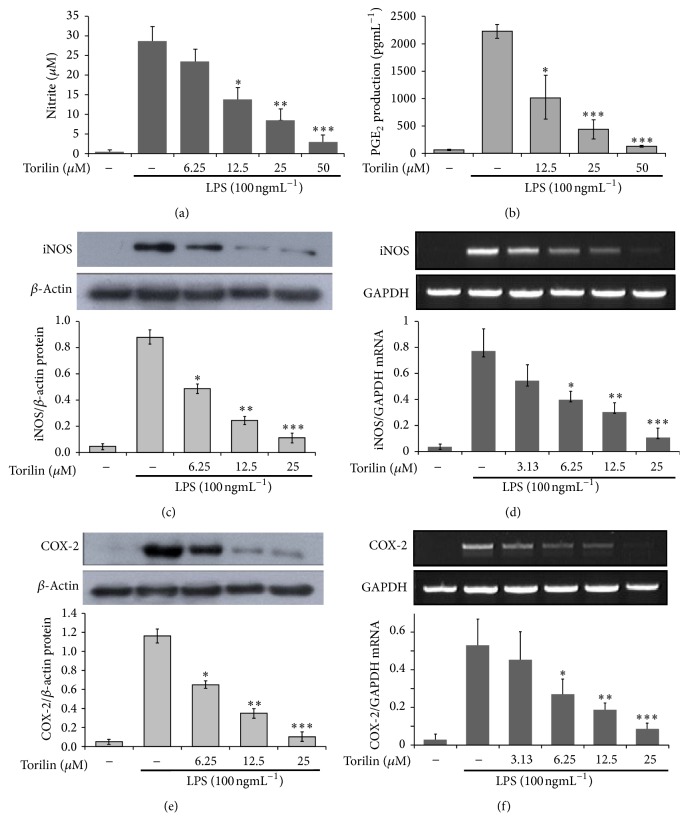
Torilin inhibits LPS-induced NO release, PGE2 secretion, and protein as well as mRNA expression of iNOS and COX-2 enzymes. RAW 246.7 macrophages were pretreated with torilin or vehicle for 30 min and stimulated with LPS for 18 or 24 h. (a) Cell culture supernatants were analyzed for nitrite release, as a measure of NO production. (b) PGE_2_ secretion in culture media was analyzed in torilin treated RAW cells as described in Materials and Methods. After 24 h of stimulation for protein expression (c) and 18 h of stimulation for mRNA expression (d) were depicted, respectively. Again, COX-2 protein (e) and mRNA (f) were determined by western blot and RT-PCR, respectively. GAPDH and *β*-actin were used as controls for mRNA and protein loading, respectively. Images are representative of 3 or 4 independent experiments. Values in bar graphs are means ± SE of at least 4 independent experiments performed in triplicate. Significance was determined using Student's* t-*test versus the control group. ^*∗*^*P* < 0.05, ^*∗∗*^*P* < 0.01, ^*∗∗∗*^*P* < 0.001 versus LPS.

**Figure 2:**
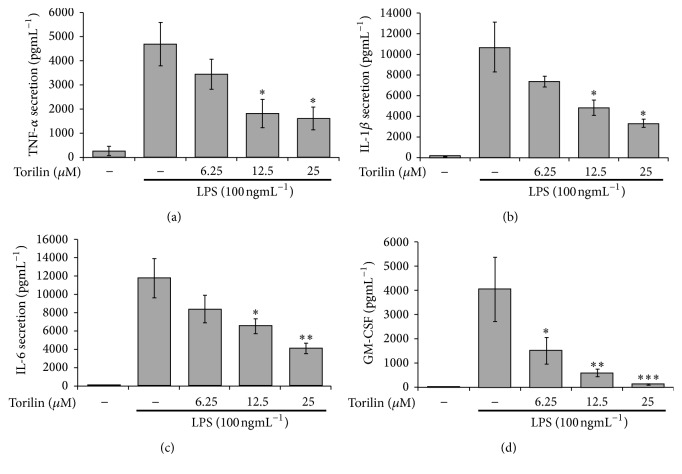
Torilin pretreatment reduces LPS-induced proinflammatory cytokine secretions into the culture media. RAW 264.7 cells were pretreated with torilin or vehicle for 30 min and stimulated with LPS for 24 h. TNF-*α* (a), IL-1*β* (b), IL-6 (c), and GM-CSF (d) were determined using ELISA. Each bar graph represents mean ± (SE) for four independent experiments. Significance was determined using Student's* t*-test. ^*∗*^*P* < 0.05, ^*∗∗*^*P* < 0.01, ^*∗∗∗*^*P* < 0.001.

Because torilin has been described as sesquiterpene with anti-inflammatory activity in BV2 cells [25], we examined the anti-inflammatory mechanisms of the compound using a suitable macrophage RAW 264.7 cell-line model. Toxicity screening showed that torilin did not exhibit cytotoxicity in RAW 264.7 (Supplementary Figure 1 in Supplementary Material available online at https://doi.org/10.1155/2017/7250968). Torilin pretreatment dose-dependently inhibited NO generation and iNOS protein and gene expressions (Figures 1(a), 1(c), and 1(d)), respectively. In addition, prostaglandin (PG)E2 and COX-2 protein and mRNA expressions were markedly suppressed by torilin (Figures 1(b), 1(e), and 1(f)). Torilin further arrested LPS-induced proinflammatory cytokines including TNF-*α*, IL-1*β*, IL-6, and GM-CSF protein secretion (Figures 2(a)–2(d)) and protein and mRNA expressions (Supplementary Figure 2A-F), respectively. These data suggested that torilin may elicit its overall anti-inflammatory effects at the level and/or upstream of inflammatory gene transcriptions.

### 3.2. Effect of Torilin on LPS-Induced MAP Kinases and PI3k-Akt Activation

**Figure 3:**
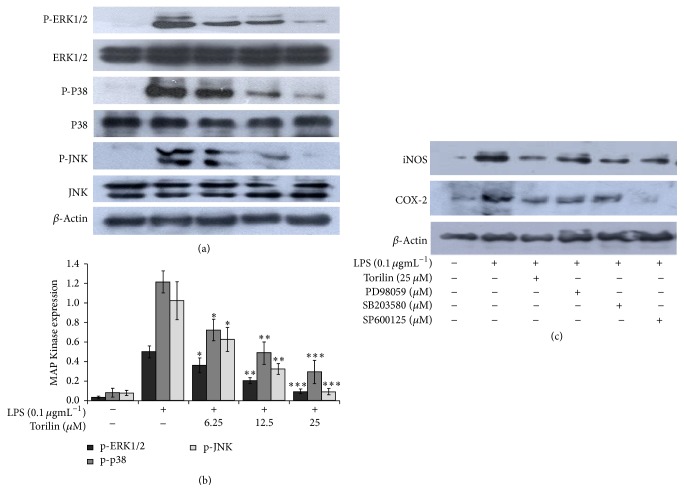
Effect of torilin on mitogen activated protein kinase (MAPK) activation. LPS-stimulated RAW 264.7 macrophages were treated with vehicle or torilin (6.25–25 *µ*M) and stimulated with LPS for the indicated periods of time. Total protein was subjected to western blot analyses using total and phospho-anti-RK1/2, p38^MAPK^, and JNK1/2 antibodies. Torilin suppressed phosphorylation of the three MAPKs (a and b). Cells were further pretreated with vehicle or PD98059 (30 *µ*M), ERK1/2 inhibitor, SB203580 (10 *µ*M), p38^MAPK^ inhibitor, or SP600125 (10 *µ*M), JNK1/2 inhibitor, with torilin and stimulated with 100 ngmL^−1^ LPS as indicated in (c). Significance was determined using Student's* t-*test versus the control group. Images are representative of 3 or 4 independent experiments. Values in bar graphs are means ± SE of at least 4 independent experiments performed in triplicate. ^*∗*^*P* < 0.05, ^*∗∗*^*P* < 0.01, ^*∗∗∗*^*P* < 0.001 versus LPS.

We examined the possible involvement of MAPK and PI3K/Akt signaling pathways in torilin mediated inhibition of inflammatory mediators. As shown in Figures 3(a) and 3(b), torilin significantly suppressed LPS-induced ERK1/2, p38, and JNK1/2 activation in a dose dependent manner while it had no effect on PI3k/Akt phosphorylation (Supplementary Figure 3). This observation was further confirmed by the suppressive effects of the respective MAP kinases inhibitors PD98059 (ERK-inhibitor), SB203580 (p38-inhibitor), and SP600125 (JNK-inhibitor) on iNOS and COX-2 expressions (Figure 3(c)). The result suggests that the anti-inflammatory effect of torilin is correlated with MAPK inactivation.

### 3.3. Torilin Inhibited I-*κ*B Phosphorylation and NF-*κ*B Activation

**Figure 4:**
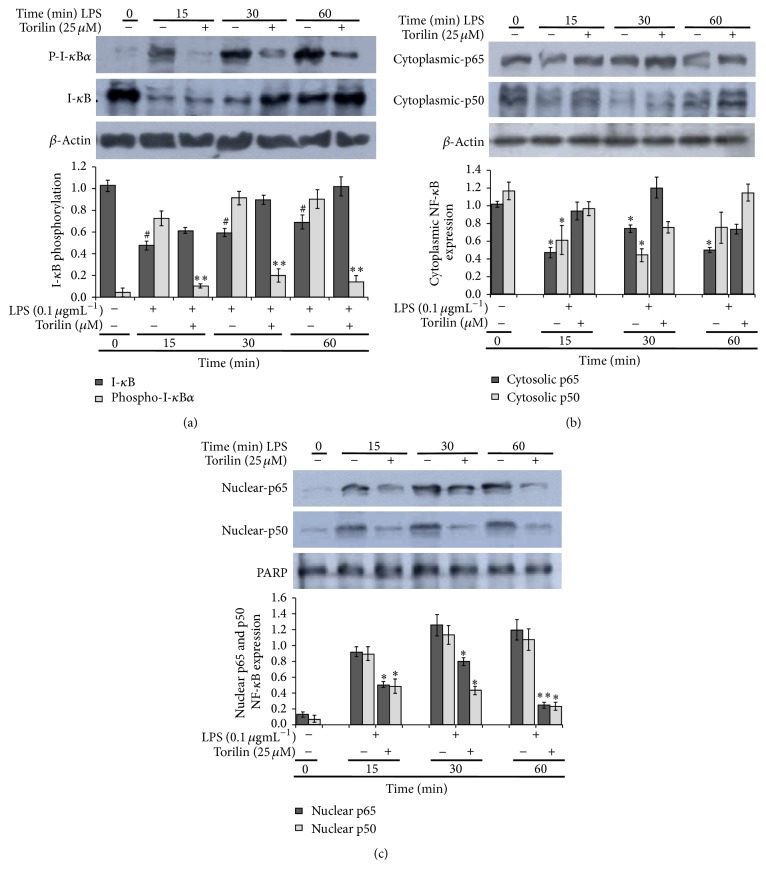
Torilin restores I-*κ*B*α* phosphorylation and degradation; thereby it inhibits p65-NF-*κ*B nuclear translocation. (a) RAW 264.7 cells were pretreated with the indicated concentrations of torilin for 30 min and incubated with LPS (100 ng/ml) for (15–60 min) time course and then assayed for the phosphorylation and degradation of I-*κ*B*α* and nuclear translocation of p65 by western immunoblot analysis as described under Materials and Methods. (b and c) RAW macrophages were treated with LPS (100 ngmL^−1^) and 25 *µ*M torilin, respectively, for the indicated time course before cytoplasmic and nuclear protein fractions were subjected to western blot analyses for p65 (upper panel) and p50 (middle panel) of cytoplasmic (b) and nuclear (c) proteins, respectively. *β*-Actin and ploy(ADP-ribose) polymerase (PARP) were used as a control for the cytoplasmic and nuclear protein loading, respectively. Images are representative of 3 or 4 independent experiments. Values in bar graphs are means ± SE of at least 4 independent experiments performed in triplicate. Significance was determined using Student's* t-*test. ^*∗*^*P* < 0.05, ^*∗∗*^*P* < 0.01, ^*∗∗∗*^*P* < 0.001. ^#^The significance of IkB degradation upon LPS activation associated with p-IkB phosphorylation in comparison with torilin treated group as analysed from western blot band size.

To further investigate the molecular mechanism involved in the torilin mediated inhibitions of inflammatory mediator and cytokine transcriptions observed in this study, we examined whether NF-*κ*B and/or AP-1 signaling pathways are involved. As shown in Figure 4(a), LPS stimulation markedly triggered I-kB*α* phosphorylation with a concurrent decrease in total I-kB expression especially at 15 and 30 min LPS stimulation. Torilin pretreatment strongly and time-dependently inhibited I-kB*α* phosphorylation and restored total I-kB depletion (Figure 4(a)). Since phosphorylation of I-kB*α* precedes degradation of I-kB*α* and subsequent release of NF-*κ*B, we also examined the effect of torilin treatment on NF-*κ*B activation and translocation.

### 3.4. Torilin Inhibits NF-*κ*B and AP-1 Nuclear Translocation, DNA Binding, and Reporter Activities

Since, without entering the nucleus, NF-*κ*B cannot regulate transcription, we investigated effect of torilin treatment on LPS-induced NF-*κ*B translocation, DNA binding, and reporter gene activity. The p65 and p50 nuclear translocation was analyzed from cytosolic and nuclear protein fractions at a given time interval. Torilin significantly reduced LPS-induced cytosolic p65 and p50 expressions (Figure 4(b)). The nuclear translocation of NF-*κ*B was markedly observed from an increased LPS-induced p65 and p50 nuclear protein expression levels that were significantly inhibited by torilin treatment (Figure 4(c)). In addition to NF-*κ*B activation (Supplementary Figure 4), immunoblotting revealed that LPS-induced AP-1 subunit (ATF-2 and c-jun but not c-fos) activation was also inhibited by torilin treatment (Supplementary Figure 5).

**Figure 5:**
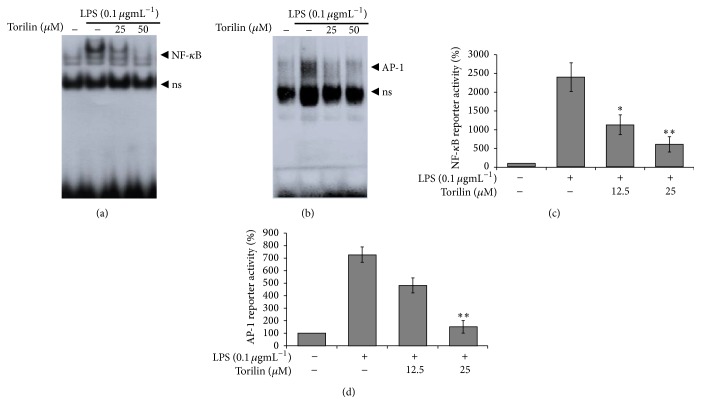
Torilin arrests AP-1 and NF-*κ*B DNA binding and reporter gene expression in LPS-stimulated RAW 264.7 cells. RAW macrophages were treated with vehicle or torilin (as indicated) and stimulated with LPS for 45 min before nuclear protein was isolated. DNA binding was analyzed using specific *γ*32P-labeled oligonucleotide probes for NF-*κ*B (a) or AP-1 (b). Specificity was demonstrated by coincubation with a 25-fold excess of unlabeled specific probe of NF-*κ*B and AP-1 for competition. Cells were transiently transfected with NF-*κ*B (c) or AP-1 (d), plasmids treated with the indicated concentrations of torilin and LPS (100 ngmL^−1^) for 6 h and assayed for CAT expression using a CAT enzyme-linked immunosorbent assay kit. Each column shows the mean ± SEM of quadruplicate determinations. Luciferase activity was normalized to *β*-galactosidase activity. Images are representative of 3 independent experiments. ^*∗*^*P* < 0.05, ^*∗∗*^*P* < 0.01, as compared with vehicle.

To examine whether torilin attenuates LPS-induced NF-kB and/or AP-1 DNA binding, we conducted EMSA analyses. As shown in Figures 5(a) and 5(b), torilin at the doses of 25 and 50 *µ*M demonstrated a selective reduction in NF-*κ*B and AP-1 DNA binding. To further investigate whether torilin attenuates promoter activities of the indicated transcription factors, we tested luciferase reporter gene transcription. Incubation of transfected RAW 264.7 cells with LPS for 6 h increased luciferase activity. However, the increased NF-*κ*B and AP-1 reporter gene activities were suppressed in torilin-sensitive manner (Figures 5(c) and 5(d)), suggesting that the compound's inhibitory effect is associated with reduced NF-kB- and AP-1-DNA binding and promoter activities.

### 3.5. Torilin Suppressed I*κ*B Kinase-*α*/*β* (IKK*α*/*β*) Activation

**Figure 6:**
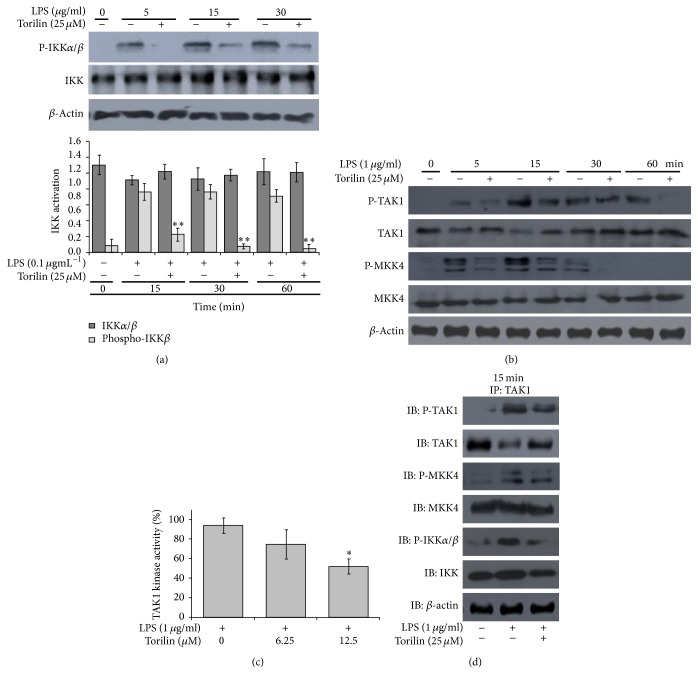
Torilin suppresses LPS-induced IKK and MAP3K complex activation and inhibits the TAK1 interaction with the components of MAPK and NF-*κ*B signaling pathways. RAW macrophage cells were incubated with torilin or vehicle for 30 min and then stimulated with LPS as indicated in the figures. (a) Lysates from torilin or vehicle treated-cells were immunoblotted to detect the activation status of IKK. (b) Immunoblots depicting the time dependent effects of torilin in TAK1, MKK4, and IKK phosphorylation. (c) The kinase activity of the TAK1 was determined after immunoprecipitates were resuspended in kinase buffer and assayed for kinase activity as described in Materials and Methods. (d) Cell lysates were immunoprecipitated by incubating overnight with anti-TAK1 antibody and then incubated with protein A-Sepharose (PAS) for 4 h at 4°C. Precipitated proteins were separated by SDS-PAGE and immunoblotted to detect P-TAK1, TAK1, p-MKK4, MKK4, p-IKK*α*/*β*, IKK, and *β*-actin. Equivalent protein loading was verified by reprobing for the respective total antibodies and *β*-actin. Images are representative of 4 or more independent experiments. Values in bar graphs are means ± SE of at least 4 separate experiments performed in triplicate. ^*∗*^*P* < 0.05, ^*∗∗*^*P* < 0.01 versus control.

Since activation of NF-*κ*B is induced by a cascade of signaling events leading to the activation of IKK complex, which in turn phosphorylates I*κ*B [28, 29], we determined torilin influence on LPS-induced IKK*α*/*β* activation. In parallel with its inhibition on LPS-induced phosphorylation and I*κ*B*α* degradation (Figure 4(a)), torilin suppressed IKK*α*/*β* activation at the indicated time course (Figure 6(a)), suggesting that LPS-induced kinase activity may be impaired by the test compound that ultimately leads to an inhibition in IKK-mediated I*κ*B*α* phosphorylation and NF-*κ*B regulated inflammatory response. However, it is worth noticing that NF-*κ*B is not the only pathway that could be modified by torilin because phosphorylation of IKK*α*/*β* is also regulated by other upstream factors such as MAPKs, including ERK, JNK, and p38. We, indeed, affirmed that torilin markedly suppressed the upregulation of these kinases after LPS stimulation (Figures 3(a) and 3(b)), suggesting that torilin affects some upstream targets as they inhibit the NF-kB and MAPK pathways simultaneously and further potentiate the suppression of inflammatory responses.

### 3.6. Torilin Inhibits LPS-Induced TAK1 Activation and Multiple Components of Downstream Signaling Pathway

Since our data showed that torilin affects both IKK*α*/*β* and MAPK downstream pathways, we thought that its inhibitory effect might involve upstream common pathway that lies between TLR4 and MAP3K complexes. Interestingly, immunoblotting analysis revealed that while torilin did not affect LPS-stimulated activation of MyD88, IRAK1, and TRAF6 (data not shown), it markedly inhibited TAK1, MKK4, and IKK*α*/*β* activation (Figures 6(a) and 6(b)). Since Shim et al. found that TNFR1, IL-1R, TLR3, and TLR4-mediated NF-*κ*B and AP-1 activation are severely impaired in TAK1 deficient cells, but not in TAK1-binding protein 1 and 2 (TAB1/2) deficient cells [30], and since torilin did not affect MyD88, IRAK1, and TRAF6 downstream of TLR4 but suppressed the signaling effects thereafter we further examined the test compound's effect at the level of MAP3K complexes. TAK1 was immunoprecipitated from LPS-stimulated untreated cells and from cells treated with torilin, as indicated in Figure 6(d). The immunoprecipitates were separated on SDS-PAGE, and the membrane was blotted with an antibody that recognizes p-TAK1, TAK1, p-MKK4, MKK4, p-IKK*α*/*β*, and IKK *α*/*β*. TAK1 precipitation increased LPS-induced p-TAK1 activation and brought down the basal TAK1 protein expression, the event of which was reversed by torilin treatment (Figure 6(d)). We were also able to detect torilin mediated inhibition of MKK4 and IKK*α*/*β* phosphorylation while the basal activities of these kinases were not affected in torilin treated and TAK1 precipitated lysates. Interestingly, torilin further inhabited LPS-stimulated TAK1 kinase activation (Figure 6(c)), suggesting torilin's upstream inhibitory effect at the level of MAP3k complex early in the signaling event through the inhibition of TAK1 activation with a marked limitation of LPS-induced NF-*κ*B and MAPKs stimulation.

## 4. Discussion

The promotion of inflammatory conditions and the initiation of the innate immune response require the release of many special effector proteins, the synthesis of which is transcriptionally regulated by inducible transcription factors that bind to the promoter regions of their respective genes. Such effectors (mediators of inflammation) include iNOS, COX-2, and cytokines (TNF-*α*, IL-1, IL-6, and GM-CSF). NF-*κ*B and AP1 are among the principal inducible transcription factors that play pivotal roles in innate immune response [31] and chronic inflammatory conditions such as rheumatoid arthritis [32]. Recently, the demand for natural products limiting induction of inflammatory mediators with minimal side effects is growing.

For decades, numerous studies have been reported to support the promise that natural products and phytochemicals could be protective against the risk of various types of inflammation and a large body of evidence indicated the anti-inflammatory effects of these compounds in terms of their action against kinases, adaptor proteins, the transcription factors, such as NF-*κ*B and AP-1, and the genes regulated by such transcription factors [8, 9, 11, 33].

Sesquiterpenes, plant-derived compounds, are reported to modulate NF-*κ*B and AP-1 transduction pathways and inhibit inflammatory mediators and inflammatory processes that help alleviate the symptoms of inflammatory diseases including arthritis [9, 11]. Since, torilin, a sesquiterpene, has been shown to possess anti-inflammatory activities in vitro and in vivo [24, 25], we, using mouse model of rheumatic arthritis, have previously reported that torilin modifies inflammatory cell and cytokine imbalances with the attenuation of the severity of arthritis [26]. We, here further, report the in vitro inhibitory effect of torilin in LPS-inducible inflammatory mediators and proinflammatory cytokines and propose the underlying mechanism of action in LPS-stimulated RAW 264.7 cells. The mediators of inflammation protein secretion and mRNA expressions revealed that torilin effectively blocked the LPS-stimulated NO generation, PGE2 synthesis, and iNOS and COX-2 protein and mRNA expressions, respectively. In addition, LPS-activated TNF-*α*, IL-1*β*, IL-6, and GM-CSF protein secretions and gene expressions are markedly inhibited by torilin treatment, suggesting that the test compound's wide-spectrum effect on inflammatory mediators might arise from its influence on the upstream common signaling pathway.

This study examined the involvement of MAPKs as a molecular target for torilin mediated inhibition of LPS-induced inflammatory mediators and proinflammatory cytokine inductions. All the three MAPKs, that is, ERK1/2, p38^MAPK^ and JNK1/2, were markedly suppressed by torilin pretreatment. Using the selective MAPK inhibitors, PD98059, SB203580, and SP600125, we further confirmed that blockade of the indicated MAPK activities by their respective inhibitors suppressed iNOS and COX-2 expressions. Moreover, torilin arrested AP1 transactivation and its subunits (ATF2, c-jun, and c-fos) phosphorylation. This observation suggests that the inhibitory effect of torilin on iNOS and COX-2 induction was at least partly regulated via MAPKs mediated AP1 transactivation. In agreement with our data, several lines of evidence documented the essential roles of MAPKs in regulating LPS-induced inflammatory responses. MAPK cascades mediate LPS-stimulated induction of COX-2 and IL-1*β* in RAW264 macrophages [34]. Likewise, ERK and p38 subgroups of MAPKs regulate iNOS and TNF-*α* gene expressions in endotoxin-stimulated glial cells [35], and p38^MAPK^ also plays role in IL-1*β* transcription [36]. Hwang et al. have reported blockade of ERK1/2 and p38^MAPK^ activities by PD98059 and SB203580, respectively, resulting in partial suppression of LPS-induced COX-2 expression [37], and monocytes treated with LPS in the presence of MEK inhibitor U0126 failed to release cytokines and PGE2 [38], supporting the notion that the MAPK pathway is critical for inflammatory response and torilin with the inhibitory potential of this pathway could be a better candidate against inflammation.

In agreement with our study, an isolated report indicated anti-inflammatory effect of torilin in BV2 cells [25]. The authors reported that torilin inhibits iNOS, COX-2, PGE2, and IL-1*β* activation and they suggested that torilin mediated inhibition of ERK or p38 but not JNK activation is one of the possible mechanisms underlying its inhibitory action on iNOS expression and NO production [25]. The discrepancy on JNK activation between the reported study and the present investigation may be due to the difference in cells used in the respective studies. In line with this, negative regulation of p38 and IKK activation by TGF-*β*-activated kinase 1 (TAK1) was observed in a cell type-specific manner [39]. However, torilin action against a wide range of LPS-induced inflammatory mediators, MAPKs, and transcription factors suggests that upstream pathway early signaling event may be affected.

Since a critical step in LPS-induced NF-*κ*B nuclear accumulation and transcriptional activity is p65 and p50 dissociation from I*κ*B protein, this study investigated effect of torilin treatment in I*κ*B and its kinase activity. Interestingly, we found that torilin inhibited LPS-induced IKK*α*/*β* activation, IkB*α* phosphorylation, and decreased p65 and p50 nuclear translocation. In line with our findings, other sesquiterpenes have been shown to inhibit activation of NF-*κ*B by preventing the degradation of I-*κ*B-*α* and I-*κ*B-*β* [13], inhibiting NF-*κ*B activation [40], and suppressing iNOS and COX-2 expression through the inactivation of NF-kB [41] or by directly binding to and inhibiting I-*κ*B kinase [14, 15].

We, in the present study, observed that torilin pretreatment suppressed MAPK and IKK mediated I-*κ*B phosphorylation, NF-*κ*B and AP1 nuclear translocation, DNA binding, and reporter transcriptional activation reflecting the ability of torilin to inhibit NF- *κ*B and AP-1 dependent inflammatory mediators and proinflammatory cytokine transcriptions. Such a strong effect of torilin on both IKK and MAPK activation in the present study suggests its role on early common upstream signaling events. However, torilin did not affect MyD88, IRAK1, and TRAF6 downstream of TLR4 but suppressed the signaling effects thereafter at the level of MAP3K complexes. Interestingly, torilin inhibited TAK1 kinase activation and phosphorylation with subsequent TAK1 mediated activation of kinases such as the I-*κ*B kinase and MAP kinases, which in turn modulate the transcriptional activities of the NF-kB and AP-1 families, respectively. However, the molecular mechanisms responsible for torilin mediated TAK1 inactivation remains to be addressed.

Since TAK1, a member of the MAPKKK family, is thought to be a key modulator of adaptive and innate immunity that mediates inducible transcription factors NF-*κ*B and AP-1 and, therefore, plays a crucial role in regulating the genes that mediate inflammation [30, 42], we thought that natural compounds such as torilin that target TAK1 activation may be an attractive strategy to treat inflammatory responses. The present study together with our previous report [26] indicated that torilin plays a crucial role against TAK1 mediated cellular response to LPS-induced and collagen-induced [26] inflammatory stimuli. TAK1 is a critical integration check point for innate and adapted immune responses upstream of MAP kinase and I-kB kinase pathways [3, 43]. In line with this study, TAK1-deficient cells failed to activate NF-*κ*B and MAP kinases in response to IL-1*β*, TNF, and TLR ligands [30]. In addition to its role in innate immunity, recent studies have defined an essential role of TAK1 in T cell receptor- and B cell receptor-induced activation of NF-kB and in the survival and development of immune cells, including mature B cells and T cells [3, 43–45]. In B cells, TAK1 is reported to be required for B cell development and NF-kB and MAPK activation induced by cytokines, TLR ligands, and BCR stimuli [3, 45].

In conclusion, we demonstrate that torilin arrested LPS-induced TAK1 kinase activation with a subsequent suppression in IKK-mediated I-*κ*B phosphorylation NF-*κ*B translocation. In addition, it attenuated TAK1 mediated MAPKs activation and AP1 transactivation. Together, the data led to suppression of NF-*κ*B and AP-1 regulated inflammatory mediator and cytokine expressions, suggesting the test compound's potential as a candidate anti-inflammatory agent.

## Supplementary Material

Supplementary Figure 1: Effects of torilin on cell viability were measured by MTT assay.
Supplementary Figure 2: Torilin treatment inhibits LPS stimulated multiple cytokine protein and gene expression.
Supplementary Figure 3: Torilin did not affect LPS-induced PI3K and Akt activation.
Supplementary Figure 4: Torilin inhibits LPS-induced NF-κB activation.
Supplementary Figure 5: Torilin attenuates ATF2, c-jun and c-fos phosphorylations.

## Abbreviations

COX-2: Cyclooxygenase-2
GM-CSF: Granulocyte-macrophage colony stimulating factor
IKK or I*κ*B kinase: Inhibitory nuclear factor kappa-B kinase
IL-1*β*: Interleukin-1*β*
IL-6: Interleukin-6
iNOS: Inducible nitric oxide synthase
MAPKs: Mitogen activated protein kinases
NF-*κ*B: Nuclear factor kappa-B
NO: Nitric oxide
PGE_2_: Prostaglandin E_2_
PI3K: Phosphatidylinositol 3-phosphate
PKB/Akt: Protein kinase B
RT-PCR: Reverse transcription PCR
TNF-*α*: Tumor necrosis factor-*α*
TAK1: TGF*β*-activated kinase 1
TAB1/2: TGF*β*-activated kinase 1 bind proteins 1 and 2.
