# Supplementary material for: Torilin Inhibits Inflammation by Limiting TAK1-Mediated MAP Kinase and NF-κB Activation
Source: Mediators Inflamm. 2017 Feb 20;2017:7250968. doi: 10.1155/2017/7250968 (PMC5337842; doi:10.1155/2017/7250968)

Supplementary Figure Legend

Suppl. Figure 1: **Effects of torilin on cell viability were measured by MTT assay**

Suppl. Figure 2: Torilin treatment inhibits LPS stimulated multiple cytokine protein and gene expression

RAW 264.7 were pretreated with torilin for 30 min and stimulated for 18 h (mRNA) or 24 h (protein) by LPS (100 $ngmL^{-1}$). Expression of mRNA was determined by RT-PCR or proteins by Western blot analysis. Dose-dependent inhibitions of TNF-α (A) and IL-1β (C) protein expressions as well as their mRNA expressions TNF-α (B) and IL-1β (D), respectively. Torilin inhibited IL-6 (E) and GM-CSF (F) mRNA expressions. GAPDH and β-actin were used as a control for RNA and protein loading, respectively. Significance was determined using Student's *t* test. Images are representative of 3 or 4 independent experiments. Values in bar graphs are means ± SE of 4 independent experiments performed in triplicate. *: $P < 0.05$, **: $P < 0.01$, ***: $P < 0.001$

Suppl. Figure 3: **Torilin did not affect LPS-induced PI3K and Akt activation**

RAW 264.7 cells were pretreated with torilin or vehicle for 30 min and stimulated with LPS (100 ng/ml) for the indicated time period. Cell lysates were analyzed by western blot with anti-PI3K and anti-Akt antibodies and the respective protein levels and phosphorylation status were determined β-Actin was used as a control for protein loading. Images are representative of 4 or experiments.

Suppl. Figure 4: Torilin inhibits LPS-induced NF-κB activation

RAW 264.7 cells were incubated with torilin or vehicle for 30 min and then stimulated with LPS for 15–60 min. Cell lysates were separated by SDS-PAGE and immunoblotted to detect NF-κB phosphorylation. Equivalent protein loading was verified by β-actin. Images are representative of three separate experiments. Values in bar graphs are means ± SE of 3 independent experiments Data are representative of three separate experiments. *P < 0.05, ***P < 0.001 vs. LPS.

Suppl. Figure 5: Torilin attenuates ATF2, c-jun and c-fos phosphorylations

RAW 264.7 cells were pretreated with the indicated concentrations of torilin for 30 min and incubated with LPS (100ng/ml) for (5–30 min) assayed for the phosphorylation of ATF2, c-jun and c-fos by western immunoblot analysis as described under 'Materials and methods'. β-actin was used as a control for protein loading. Images are representative of 4 or more independent experiments.

Supplemental Figure

Supplementary Fig. 1

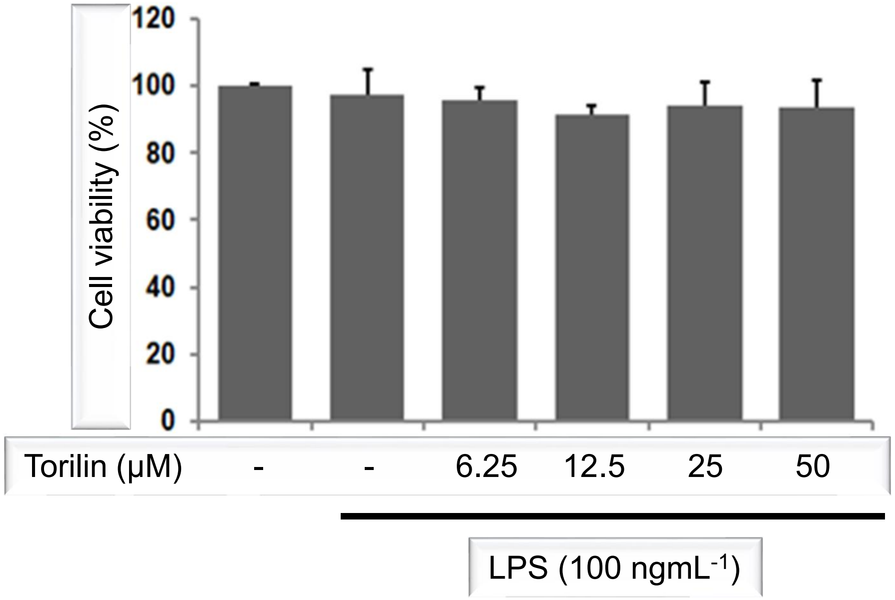

Supplementary Fig. 2

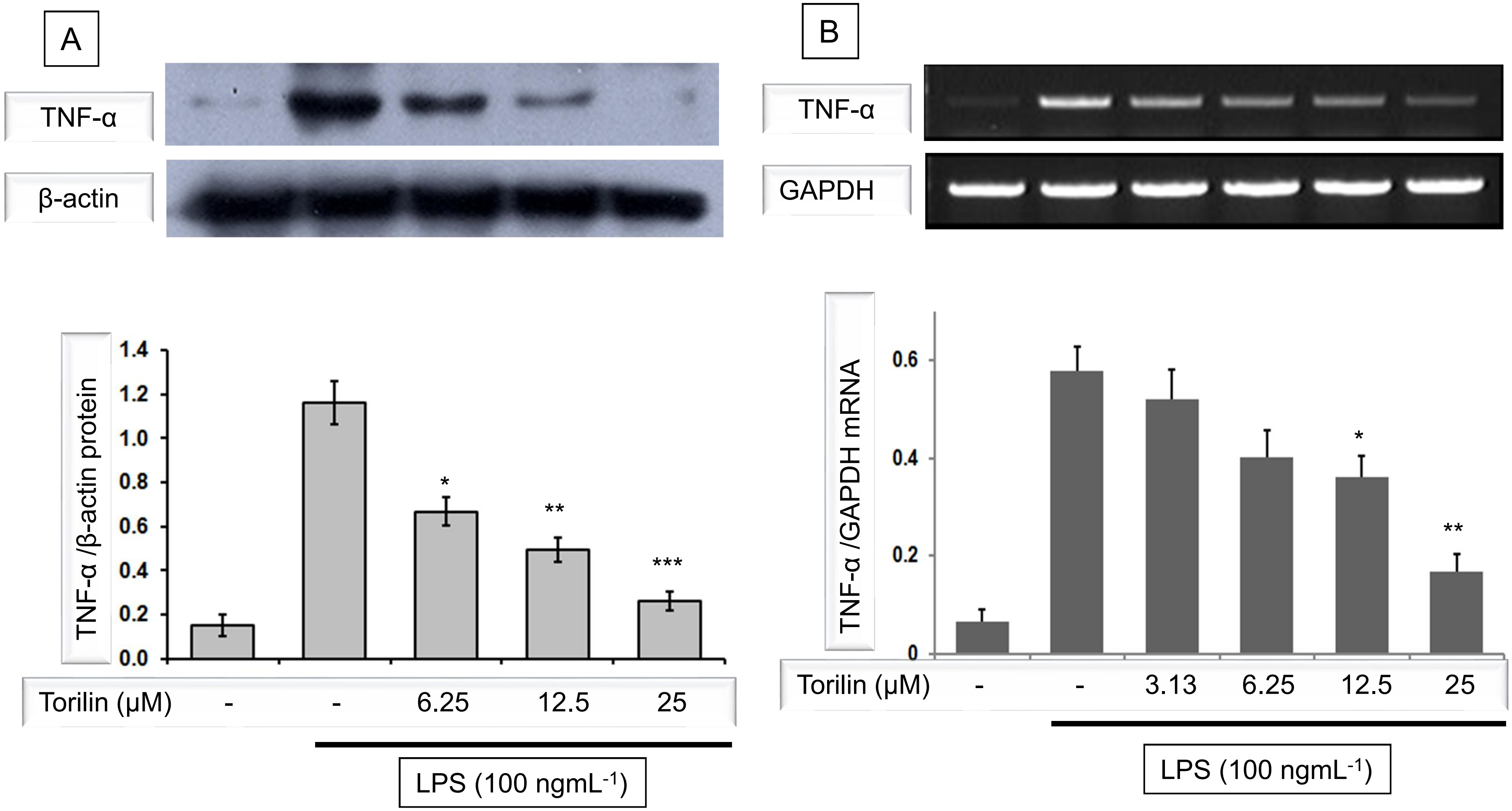

Supplementary Fig. 2

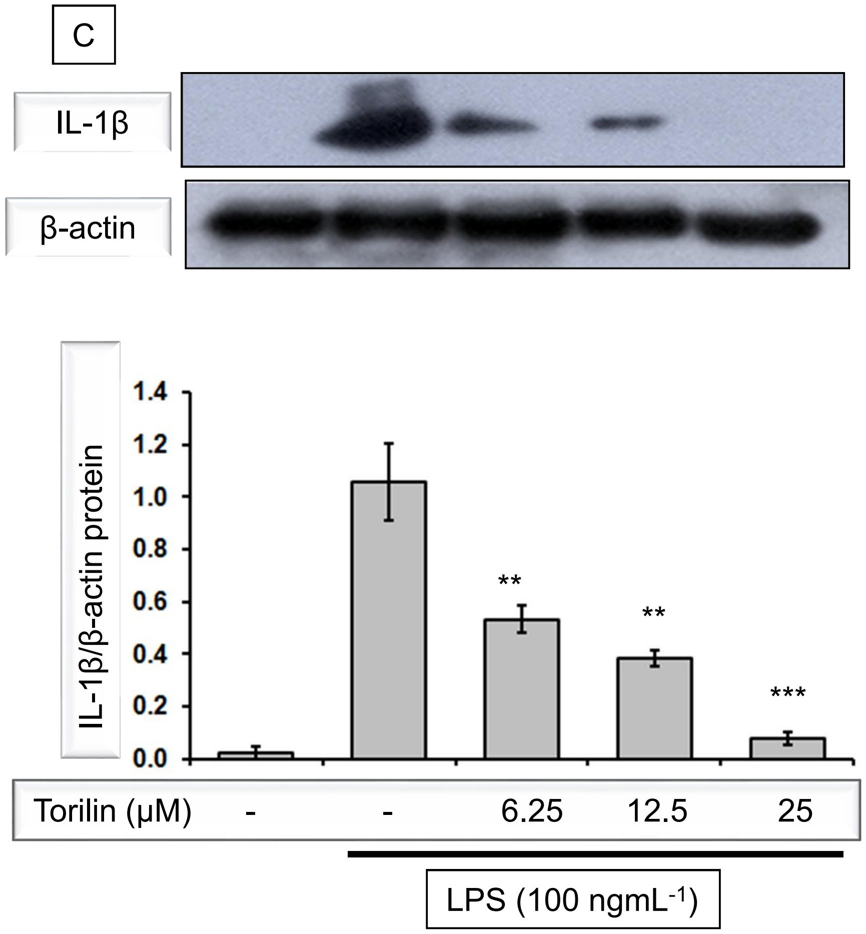

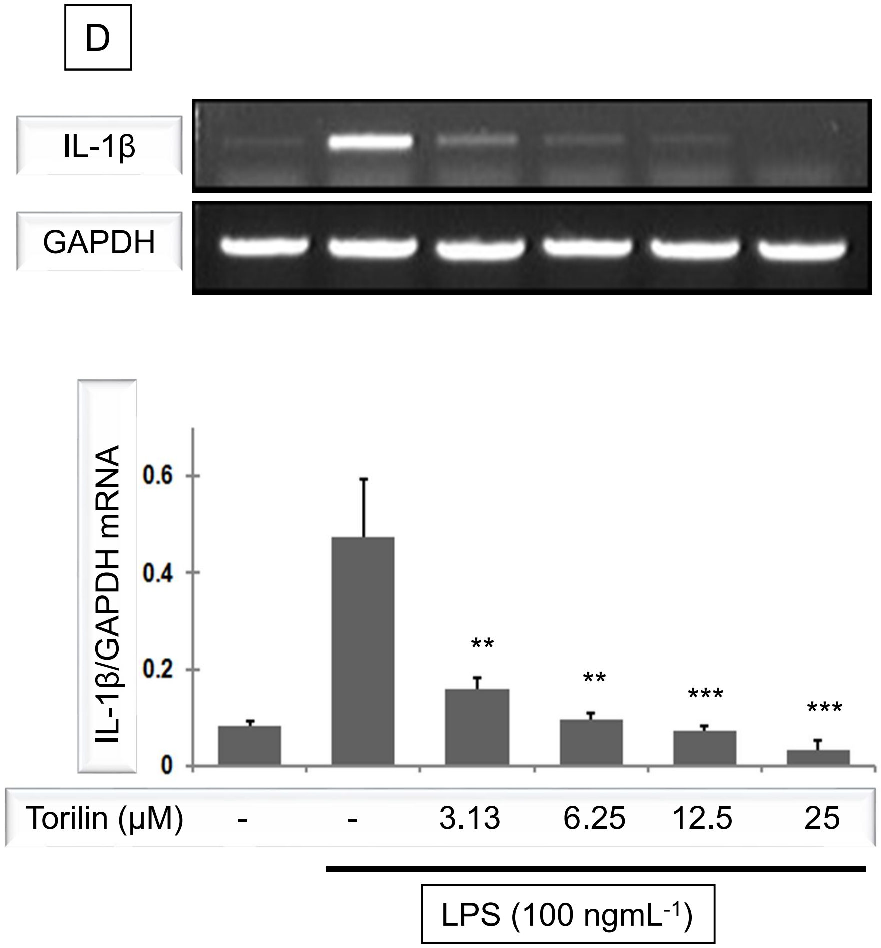

Supplementary Fig. 2

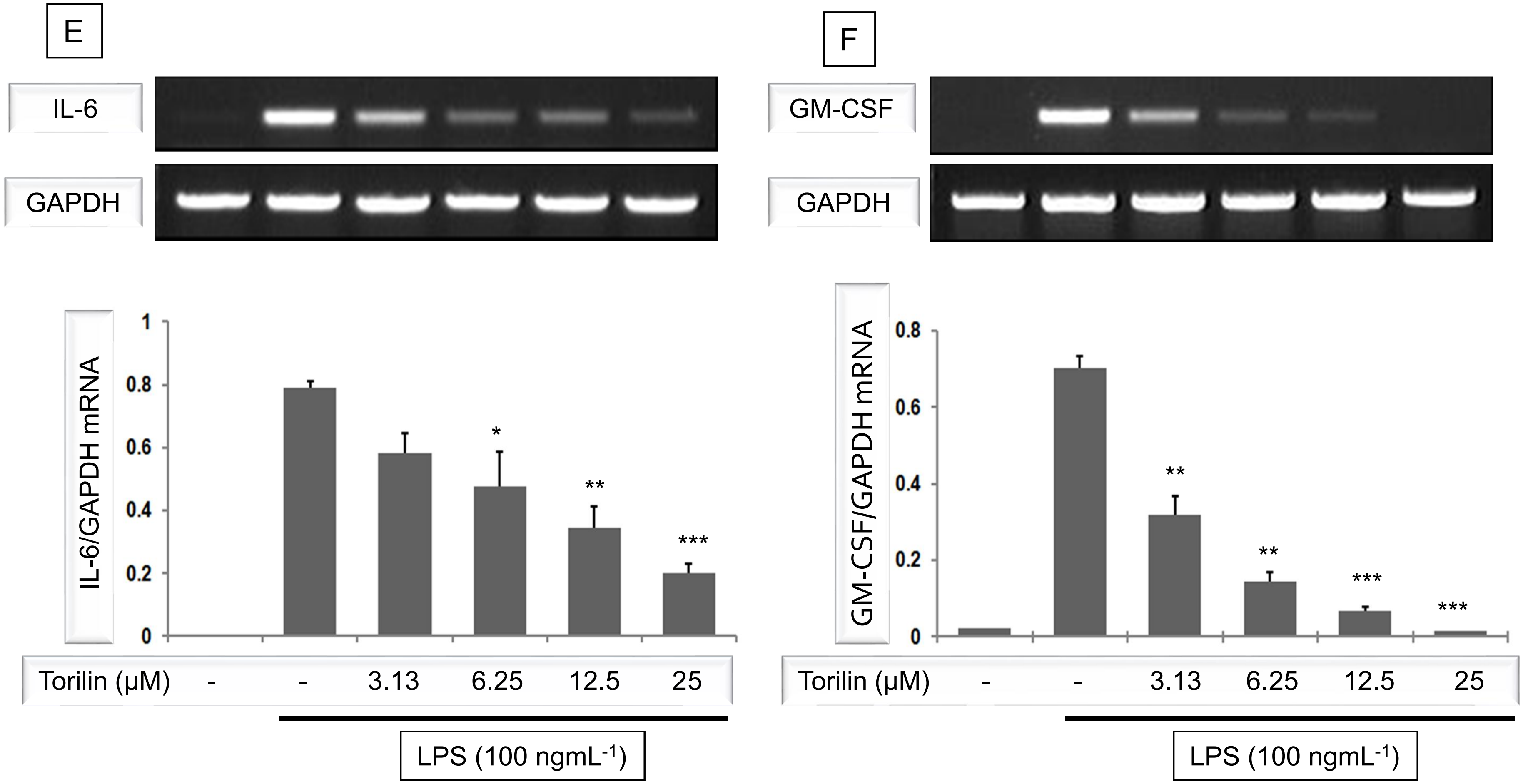

Supplementary Fig. 3

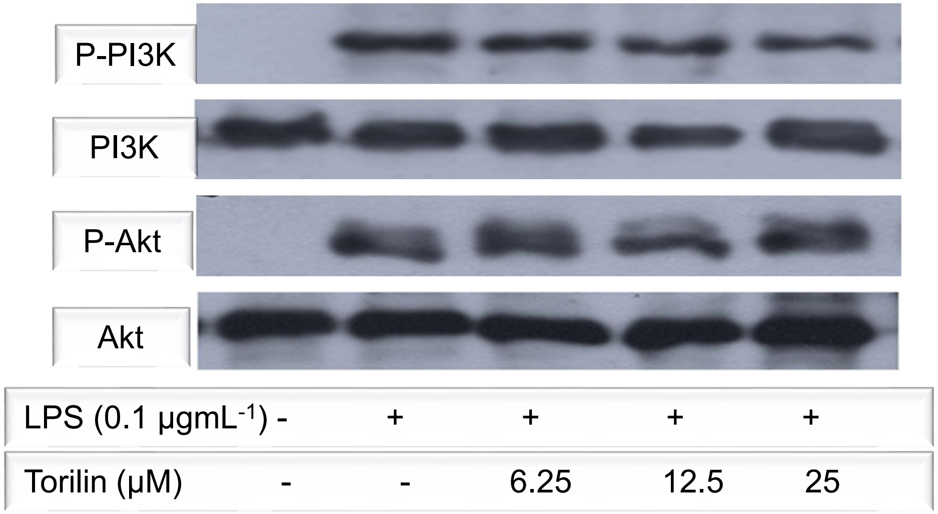

Supplementary Fig. 4

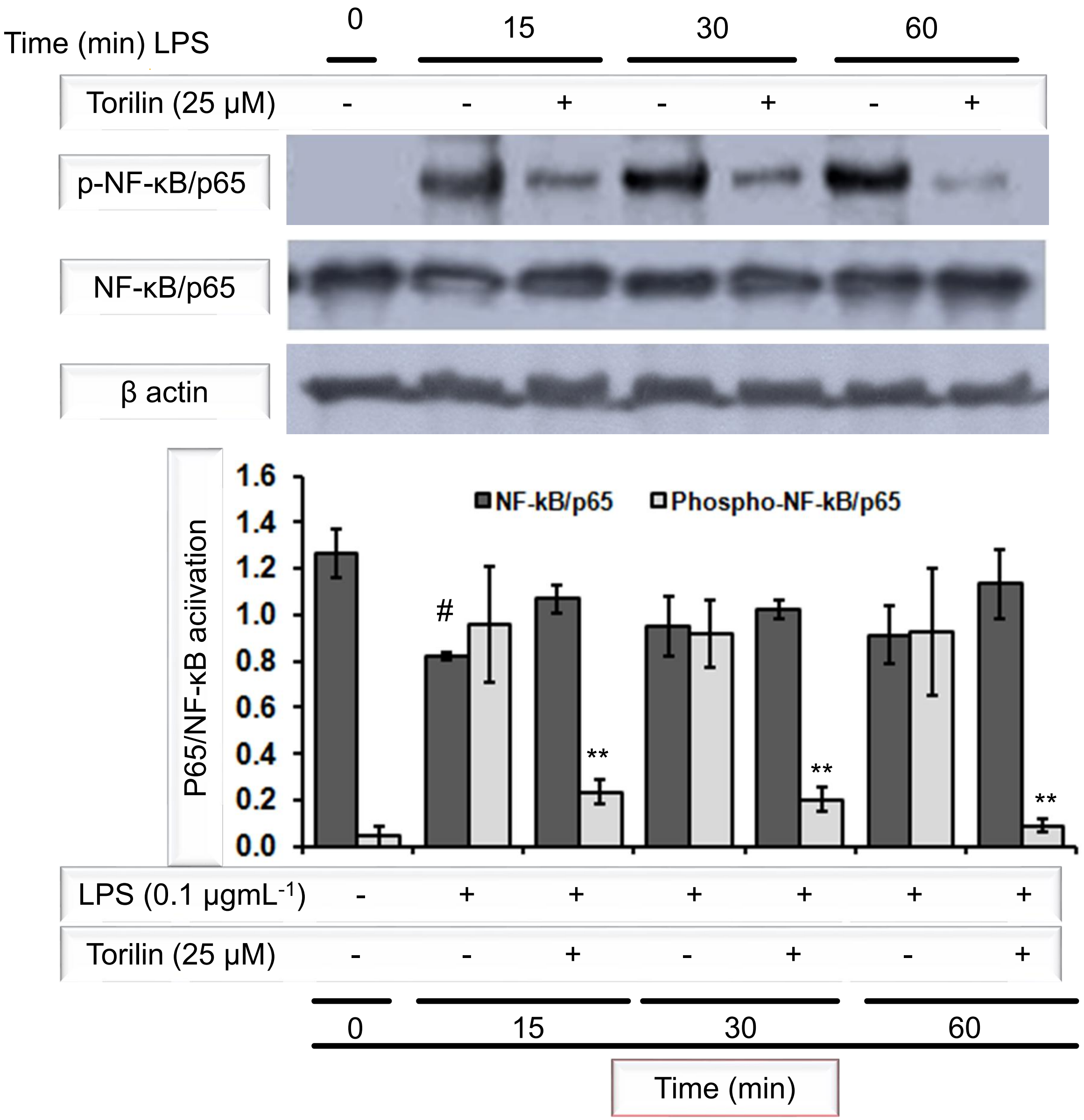

Supplementary Fig. 5

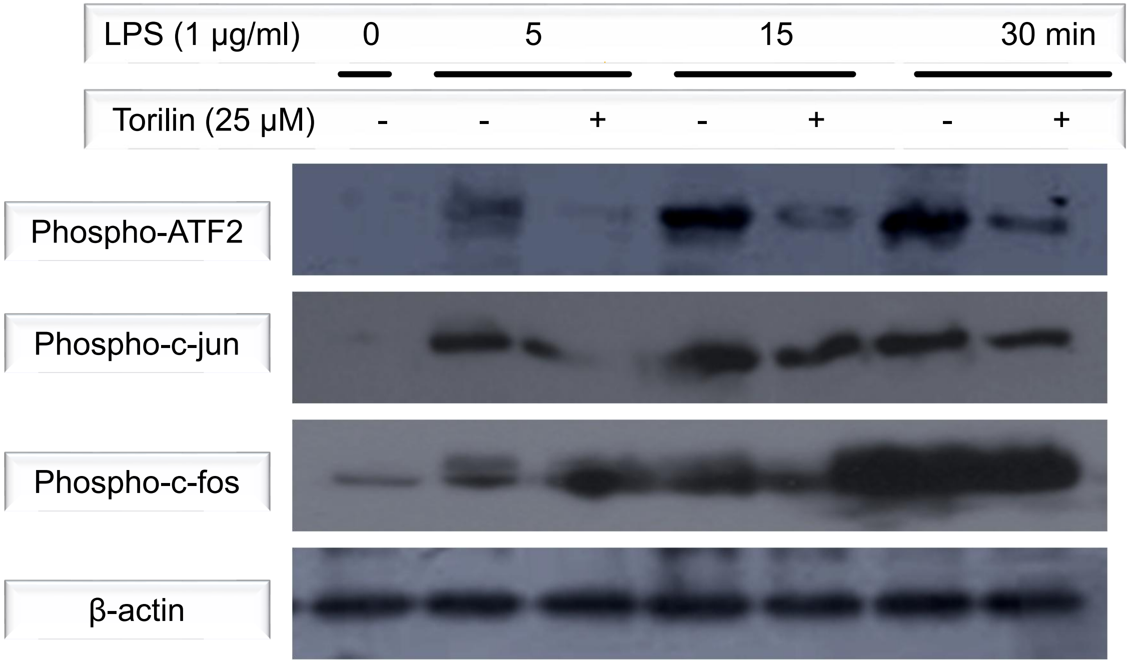

Supplement: Supplementary file 1 — Supplementary Figure 1: Effects of torilin on cell viability were measured by MTT assay. Supplementary Figure 2: Torilin treatment inhibits LPS stimulated multiple cytokine protein and gene expression. Supplementary Figure 3: Torilin did not affect LPS-induced PI3K and Akt activation. Supplementary Figure 4: Torilin inhibits LPS-induced NF-κB activation. Supplementary Figure 5: Torilin attenuates ATF2, c-jun and c-fos phosphorylations. [file 7250968.f1.pdf]
